# Electromyographic characteristics of pelvic floor muscles in women with stress urinary incontinence following sEMG-assisted biofeedback training and Pilates exercises

**DOI:** 10.1371/journal.pone.0225647

**Published:** 2019-12-02

**Authors:** Daria Chmielewska, Magdalena Stania, Katarzyna Kucab–Klich, Edward Błaszczak, Krystyna Kwaśna, Agnieszka Smykla, Dominika Hudziak, Patrycja Dolibog

**Affiliations:** 1 Department of Physiotherapy Basics, Faculty of Physiotherapy, Jerzy Kukuczka Academy of Physical Education, Katowice, Poland; 2 Department and Faculty of Medical Biophysics, Medical University of Silesia, Katowice, Poland; 3 Health Center, Department of Gynecology and Obstetrics, Mikołów, Poland; University of Thessaly, GREECE

## Abstract

The aim of this study was to compare the effect of pelvic floor muscle training with surface electromyographic (sEMG) biofeedback (BF group) and Pilates exercises (P group) on the bioelectrical activity of pelvic floor muscles in women with stress urinary incontinence. The other aim aim was to compare changes in voiding diaries and scores on quality of life questionnaire against baseline values and between the groups. Women in the BF group (n = 18) participated in pelvic floor muscle training with sEMG biofeedback; the P group (n = 13) participated in basic level Pilates workouts. Both protocols were continued for eight weeks. Voiding diary, quality of life and electromyographic characteristics of the pelvic floor muscles were assessed at the three-time points: at baseline, after eight weeks’ training, and at month six post-training. The sEMG activity of the pelvic floor muscles was tested during five trials in two positions. There was no marked improvement in bioelectrical activity of the pelvic floor muscles during contraction following training with sEMG biofeedback or Pilates exercises. Following eight weeks of sEMG biofeedback training, a decrease was noted in resting bioelectrical activity of pelvic floor muscles and during relaxation after sustained contraction but only in supine-lying. No such effect was observed in the Pilates group. In the BF group, the number of incontinence episodes after end of treatment (timpepoints: 1vs. 2) and at six month follow-up (timpepoints: 1vs. 3) decreased by 68.5% and 89.3%, respectively. The respective values in the P group were 78.6%, and 86.4%. The intergroup differences did not reach the level of statistical significance. As regards the quality of life, the questionnaire demonstrated that Pilates exercises had significantly better effects compared to biofeedback training both at the end of the eight-week exercise program and (p = 0.003) and at six month follow-up (p = 0.0009). The International Consultation on Incontinence Questionnaire—Short Form (ICIQ- SF) showed comparable efficacy of Pilates exercises and training with sEMG biofeedback. Intragroup improvements in micturition frequency, incontinence (leakage) episodes, and nocturia frequency were comparable. Alleviation of urinary incontinence symptoms was comparable in both groups, whereas the improvement in the quality of life was more notable in the Pilates group. The obtained results failed to demonstrate the superiority of any of the two methods regarding the bioelectrical activity of pelvic floor muscles in patients with stress urinary incontinence.

## Introduction

The most common types of female urinary incontinence are stress urinary incontinence, defined according to ICS, as involuntary leakage from the urethra, synchronous with exertion/effort, sneezing or coughing. The recommended non-surgical and non-pharmacological treatment methods of urinary incontinence include pelvic floor muscle training (PFMT) and biofeedback [1]. Biofeedback uses a muscle activity registering apparatus to provide information on the quality of movement activities. The information on task performance is communicated to the patient in a visual or acoustic format.

Surface electromyography (sEMG) is considered an acceptable tool for real-time evaluation of pelvic floor muscle (PFM) contractions [2, 3] and for the assessment of PFM function [4] by identification of the PFM motor unit action potential [5]. Electrical signals from the muscles are generated by the recruitment of motor units during contraction; a correlation has been observed between muscle strength and the activation of motor units [6]. The bioelectrical activity of a muscle can be monitored as a representation of muscle function [7]. Grape et al. showed good to high retest reliability of pelvic floor muscles EMG [8]. The results of studies show that differences might be expected regarding the EMG amplitude obtained in supine and standing positions. Incontinent subjects of Capson et al. exhibited significantly higher resting PFM activity in the standing compared to supine position. Resting PFM activity was higher in the standing hypolordotic posture as compared to the normal and hyperlordotic postures [9]. Standing is associated with changes in intraabdominal pressure which might cause stress leakage [1]. Furthermore, incontinent women had lower PFM activities, especially in the standing position, related to age and vaginal deliveries [10].

Dannecker et al. [11], Bertotto A et al. [12], Aukee et al. [13, 14] used sEMG to assess treatment progress, and the increases in muscle activity expressed as amplitude increase (in microvolts). These studies have shown that the biofeedback component in the group with PFM training resulted in a significantly greater increase in sEMG amplitude of the PFM compared to the group on PFM training alone.

Despite considerable interest in the effects of biofeedback-assisted exercises on urinary incontinence [11, 15] and other pathologies, the results of studies on the complementary effect of biofeedback in pelvic floor training programs remain inconclusive.

In another study observed significantly higher EMG microvolts amplitude (only during 3-s contractions) in the pelvic floor muscles in the experimental group (healthy nulliparas in uncomplicated pregnancies) compared to control group after the exercise program with EMG biofeedback [16].

The literature provides examples of significantly improved PMF function and strength in women after pelvic floor biofeedback training compared with non-feedback groups [12, 17]. However, some researchers failed to find evidence for better efficacy and effectiveness of pelvic floor muscle training enhanced by adjunctive therapies such as biofeedback [18]. A randomized study of Culligan et al. revealed comparable improvements of pelvic floor muscle strength after a Pilates exercise program and pelvic floor muscle training. Pelvic floor distress inventory scores also improved from baseline but not between groups [19]. The main idea of Pilates is to coordinate breathing and movement in order to engage the core muscles, i.e., the transversus abdominis, pelvic floor muscles, multifidus as well as the diaphragm; this coordination maintains the rhythm of the movement sequence [20]. The interaction between the pelvic floor muscles at the bottom of the abdominopelvic cavity, lumbar multifidi muscles, diaphragm and abdominal wall musculature [21] might be utilized in Pilates exercises. The Pilates method may constitute an attractive alternative for prevention or treatment of pelvic floor dysfunction [22]. A systematic review of randomized controlled trials by Bo et al. does not provide evidence of the superiority of Pilates exercises, abdominal training, or the Paula method over pelvic floor muscle training with respect to reduction of urinary incontinence symptoms [23] but shows that only two studies deal with the effect of Pilates exercises on pelvic floor muscle function in healthy and incontinent women.

We did not come across a paper comparing the effects of Pilates exercises and sEMG biofeedback in women with stress urinary incontinence. Considering the above, we decided to employ surface electromyography to assess the activity of the pelvic floor muscles before and at two timepoints after participating in a program of Pilates exercises and pelvic floor muscle training with sEMG biofeedback.

A hypothesis was formulated that, in women with stress urinary incontinence, sEMG biofeedback-assisted pelvic floor training (BF group) and Pilates exercise program (P would lead to different increases in PFM sEMG activity and that changes in the voiding diary and quality of life questionnaire would differ between the study groups and when compared to baseline. We also hypothesized that sEMG amplitude obtained in supine and standing positions at baseline (timepoint 1), immediately after both eight -week training protocols (timepoint 2), and at six months post-training (timepoint 3) would be different.

## Materials and methods

Women 45 years of age and older referred to a specialist clinic with symptoms of urinary incontinence were assessed by a clinician. Those diagnosed with stress urinary incontinence were invited to participate in the pelvic floor muscle training with physiotherapists. The following criteria of inclusion were used: stress urinary incontinence symptoms (urine leaks on coughing or sneezing or when walking or exercising, small urine leaks), at least 2 urine leakages per week, mild or moderately severe urinary incontinence as indicated by the International Consultation on Incontinence Questionnaire—Short Form (ICIQ-UI SF) [24, 25] and moderate symptom distress by the Urogenital Distress Inventory (UDI 6). The exclusion criteria included: previous therapy or surgery for urinary incontinence, urge incontinence, neurogenic urinary incontinence, uncontrolled diabetes, neurological abnormalities, urinary tract infection, cerebral stroke, advanced neoplastic disease, acute inflammatory conditions and infections, past or present injuries within the pelvis, hip joint or spine area, pelvic organ prolapse POP-Q Stages 3 and 4, previous experience with pelvic floor muscle training, Pilates or sEMG biofeedback. Following preliminary recruitment at the specialist clinic, the candidates contacted one of the researchers on the phone to declare whether they were willing to participate in the study. They also decided on the type of PFM training (sEMG biofeedback or Pilates exercises) they wanted to join. Due to the nature of the intervention and the lack of centers for pelvic floor muscles rehabilitation in our country blinding of participants and physiotherapists was not feasible. Measurements were performed under standard testing conditions, the same for all subjects. Candidates were presented with a comprehensive description of the aim and methods of the study and gave their written consent to participate. This study carried out between September 2014 and February 2018. The study protocol was approved by the Bioethics Committee at the Academy of Physical Education in Katowice, Poland (4 / 2012). All subjects gave written informed consent in accordance with the Declaration of Helsinki.

In line with the purpose of our trial, sEMG was used to test changes in the activity of the pelvic floor muscles at baseline, after eight weeks of biofeedback training and Pilates exercise program as well as at six months post-training.

### Study outcomes

1. Intra- and intergroup comparisons of changes in the activity of the pelvic floor muscles as per electromyographic recordings made at three timepoints in standing and supine positions, and between positions.
2. Intra- and intergroup comparisons of changes in symptom severity (mean number per 3 days): incontinence episodes, micturition, and nocturia (at three measurement timepoints),
3. Intra- and intergroup comparisons regarding quality of life improvement as measured with the Kings Health Questionnaire (at three-time points).

### Procedure

The pelvic floor muscle training with sEMG biofeedback and Pilates exercise programs both consisted of 24 sessions (i.e., three times weekly for eight weeks [26, 27] preceded by three instruction sessions (the same for both study groups). EMG biofeedback training was provided during individual sessions with a physiotherapist; Pilates exercises were carried out in groups consisting of three participants and supervised by a physiotherapist Pilates instructor. It should be noted that sEMG training and Pilates exercise providers were not those who subsequently analyzed pelvic floor electromyography recordings at all three timepoints.

#### Instruction session 1

Participants were asked to state their age, weight, height, medicaments, and obstetric history (number of births and cesarean deliveries, menopausal status, hormone therapy). All women were introduced to bladder diary, received standardized self-report measures to assess symptoms of incontinence and their impact on the quality of life. They were also requested to fill in the questionnaires before the next appointment. Quality of life was assessed utilizing the King's Health Questionnaire (KHQ) that yields two scores. The Part 1 score was calculated as the sum of normalized scores from questions 1 and 2. The sum of questions 3 to 8 was calculated as Part 2 score [28].

Based on voiding diaries, the mean number of leakage episodes per 3 days [29], micturition and nocturia frequency were determined before and after training and at six -month follow-up. Participants were instructed to complete their voiding diaries immediately after urination. They were educated about the anatomy and physiology of lower urinary tract and pelvic floor and gave their consent to sEMG examination using a vaginal electrode.

#### Instruction session 2

The participants were instructed in correct contraction and relaxation of the pelvic floor muscles and observed, on the computer monitor, how sEMG signals of PFM were recorded [30]. The an endovaginal probe (Everyway Medical Instruments Co, PR-02A) inserted following the application of a small amount of lubricant with the sensors positioned laterally in the vagina was used to record he sEMG of the pelvic floor muscles.

The reference surface electrode (silver/silver chloride) was placed over the right anterior superior iliac spine. The next two bipolar self-adhesive electrodes were located on the right side along muscle fibers of the rectus abdominis, transversus abdominis, and gluteus maximus in order to record their activity during voluntary pelvic floor muscle contraction. The same investigator gave verbal instruction to the study participants and made sEMG recordings. The sEMG procedures were compatible with the SENIAM standards for surface EMG [31].

#### Instruction session 3

During the first phase of sEMG testing, each participant was instructed to perform maximal voluntary contractions (MVCs) of PFM as forcefully as possible for about 5 seconds. Three attempts were made with 40-second rests between each contraction [32]. MVCs were used as reference values for PFM [33]. MVC was recorded in the supine position, with the hip and knee joints flexed at 30° and 90°, respectively. Afterward, the participants took part in five trials designed to determine pelvic floor muscle activity as described by Glazer [6, 34, 35].

SEMG evaluation parameters: *Trial 1*–10-second resting muscle activity. Parameter measured was: average peak (%MVC), mean amplitude (%MVC); *Trial 2*–5 repeated short (quick-flick) contractions with a 10-second pause between each contraction. Parameters measured were *average peak* (%MVC) (the average of all five local peaks, calculated when the amplitude exceeds the threshold level predetermined as 50% between minimum and maximum amplitudes of a particular recording) and *average mean* (%MVC) (mean amplitude of the active sEMG portions, calculated when the amplitude exceeds the threshold level predetermined as 50% between minimum and maximum amplitudes of a particular recording); *Trial 3*–5 repetitions of 10-second voluntary contractions with 10 seconds of rest in between. Parameters measured were *peak amplitude* (%MVC) and *mean amplitude* (%MVC) (see above). *Trial 4*- a sustained 60-second contraction. Parameters measured were: median frequency (Hz), mean frequency (Hz) and RMS sEMG (μV). The root mean square (RMS) values were calculated using a 100ms sliding window. *Trial 5*–10-s relaxation immediately after the 60-s contraction (parameters measured were mean amplitude (%MVC) and average peak (%MVC) (see above).

sEMG recordings were obtained while lying supine and standing [36]. Testing positions were assigned randomly with 5-minute breaks between each measurement to avoid muscle fatigue. Prior to the measurements, the participants were asked to fully void to minimize the impact of bladder filling on the tonic activity of the pelvic floor muscles [37].

#### SEMG biofeedback training in group BF (weeks 1–8, training session 1–24)

Training sessions with visual biofeedback (contractions were displayed on the computer screen) were held over eight weeks [26, 27]. Exercises (phasic fiber recruitment) and endurance exercises (tonic fiber recruitment) were performed three-times a week (a total of 24 training sessions). The duration of one session was 30–50 minutes. Exercises were supervised [12, 38] by another person than that who performed the sEMG evaluation. The Myo Trace 400 (Noraxon U.S.A. Inc.) devices were also used for biofeedback training.

At the outset of sEMG biofeedback training, each participant performed a maximal voluntary contraction of the pelvic floor muscles. The MVCs values were used as a reference threshold for the training. To improve muscle strength, the PFM training was preset at 80% of the MVC and performed as contraction/relaxation units (3 seconds/6 seconds). The number of short contractions increased progressively every week (from baseline to 8^th^ week) from 21 units (7 contraction/relaxation in 3 series with 3-minute interval between series) to 60 units (12 contraction/relaxation units in 5 series with 3-minute interval between series). Endurance training consisted of 45 (3 series, 15 contractions) to 120 (8 series, 15 contractions) contraction/relaxation units at 60% MVC with 90-second interval between each series. The duration of contraction/relaxation units was 5 seconds (weeks 1 through 4) and 10 seconds (weeks 5 through 8) [12, 15].

#### Pilates exercises in group P (weeks 1–8, training session 1–24)

Pilates exercise workouts were carried out for 8 weeks (basic level), 3 sessions of 40–50 minutes a week (a total of 24 training sessions). There were 3 participants in each training group exercising under the supervision of a certified Pilates instructor. The Pilates group performed a Pilates exercise program with the addition of voluntary PFM contraction.

Mats, therapeutic balls and sensorimotor pillows were used. The women were instructed to perform a PFM and transversus abdominis contraction during exercises. The exercise protocol is presented in S1 Table.

All participants (BF and P) were also instructed to integrate PFM contractions into daily activities and were trained to add the Knack maneuver with coughing, and sneezing.

### Signal sEMG processing

The device we used in the study (Myo Trace 400 Noraxon U.S.A. Inc., with a 16-bit analog to digital (A/D) converter with an anti-aliasing filter set to 500 Hz) recorded the sEMG activity of the PFM and the surrounding muscles. The level of common mode rejection ratio was higher than 100 dB at 60 Hz, input impedance was >100 MΩ and amplifier gain 500.

The raw full-wave sEMG signals were band-pass filtered with cut-off frequency 10-500Hz rectified and smoothed using the root mean square technique with a 100 ms sliding window. Amplitude sEMG signals of the pelvic floor muscles in standing and sitting were normalized to the MVC performed in the supine-lying [32].

### Statistical analysis

We assumed the probability of a type I error alfa rate of 0.05, maximum acceptable value for a type II error beta 0.20, the target power 1-beta of 0.80 and a 25% minimum significant difference between the means of the parameters studied. The resultant minimum sample size was 20 participants in each group.

Given that the distribution of results in a sample was unimodal and skewness and flatness were lower than 2.5, the arithmetic mean and standard deviation were selected as the most appropriate to assess central value and dispersion (of sEMG variables). The Shapiro-Wilk test was used to check the data for normal distribution. Variance homogeneity was assessed with the Levene’s test. Since several variables did not fit the normal distribution, the nonparametric tests were used. When the same variable was measured several times (k> = 2) in the same subject, but under different conditions, data from the voiding diaries, ICIQ–UI SF, the King’s Health Questionnaires and sEMG records were analyzed with the Friedman ANOVA by ranks. Post-hoc comparisons were performed using the Friedman rank sum test. The Chi-square test was used for intergroup comparisons of nominal variables and the Mann-Whitney U test for variables that did not meet the assumptions of normal distribution and variance homogeneity (age, mean BMI). The Mann-Whitney U test was used to compare data from the voiding diaries (micturition and nocturia frequency, leakage episodes), ICIQ–UI SF, the King’s Health Questionnaires and sEMG between groups BF and P. The level of statistical significance for all analyses was set at p<0.05. Data analysis was performed with Statistica, version 13.

## Results

Originally, the BF group comprised 20 women; two were lost to follow-up due to a long distance between the place of residence and our laboratory (timepoint 2). The remaining 18 women (mean age 52.9; SD 4) were subject to analysis.

The P group initially comprised 20 women. Three withdrew from the study due to a long distance between the place of residence, and our laboratory where bioelectrical activity of pelvic floor muscle was evaluated, another two resigned because of an illness in the family (timepoint 2). Due to artifacts in sEMG signal recordings of two participants, 13 (mean age 51.5; SD 5.2) women were included in the analysis.

**Fig 1:**
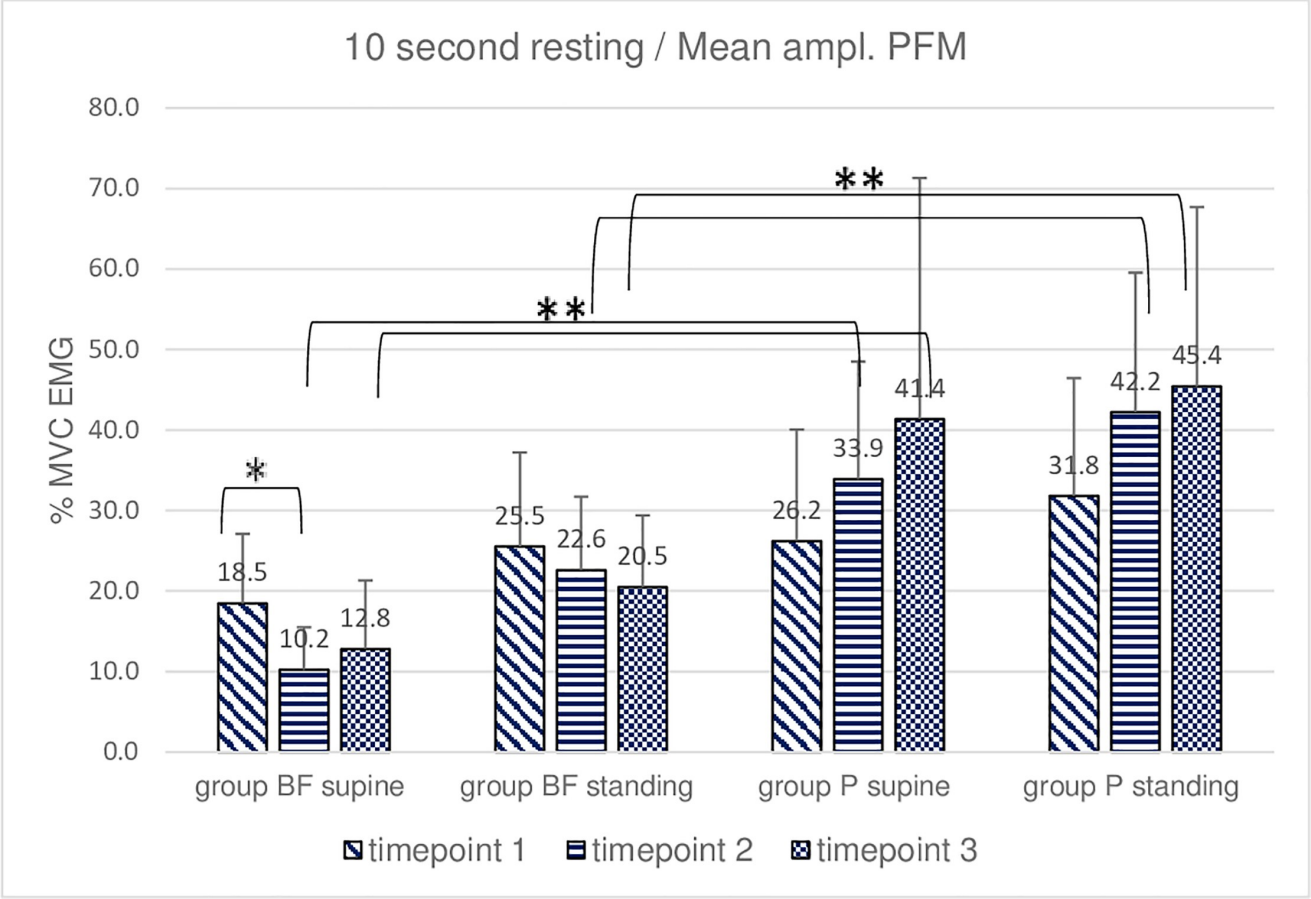
Intra- and intergroup analysis of sEMG mean %MVC amplitudes of the resting activity of pelvic floor muscles; p* Post-hoc Friedman's rank sum test; p** Mann- Whitney U test.

**Fig 2:**
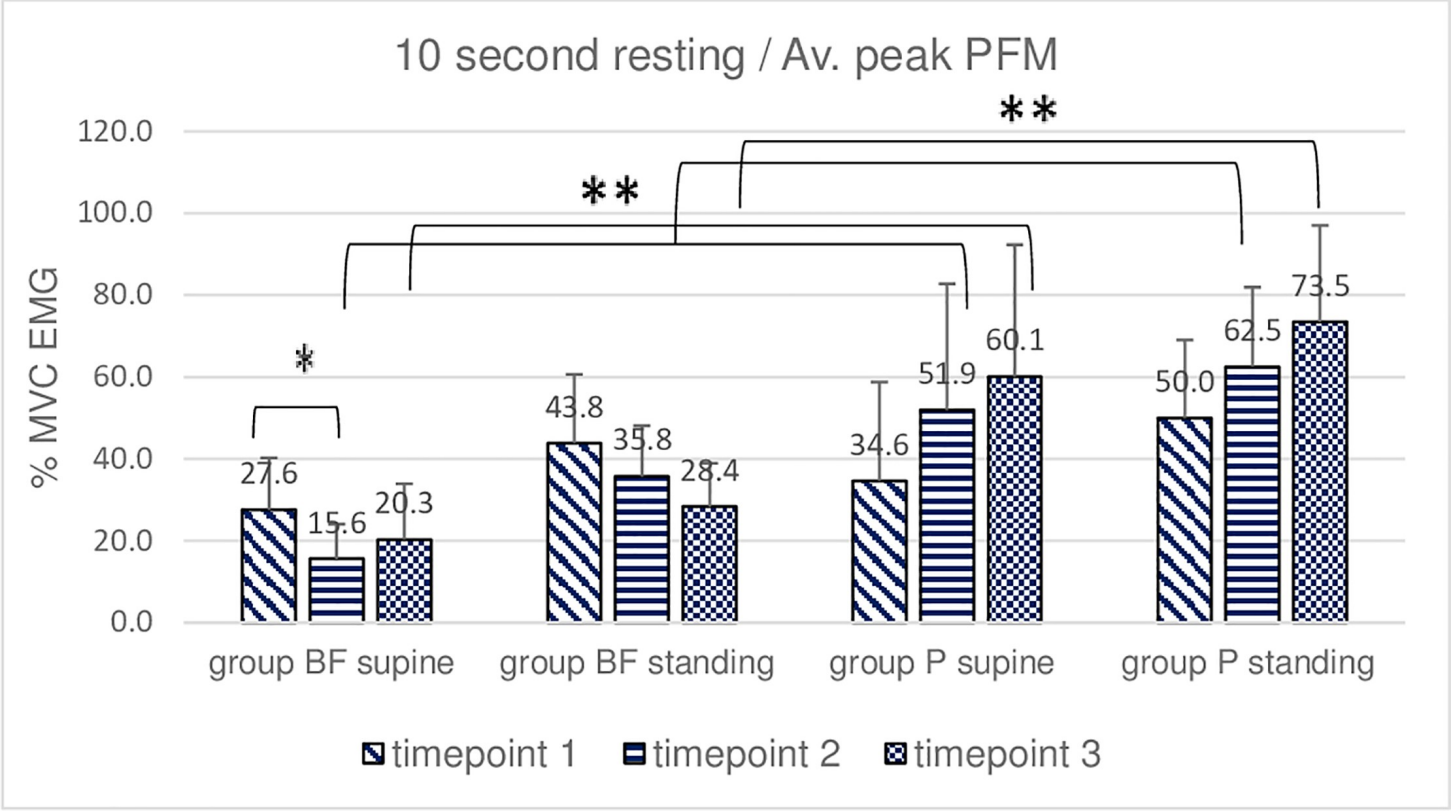
Intra- and intergroup analysis of sEMG average peak %MVC amplitudes of the resting activity of pelvic floor muscles; p* Post-hoc Friedman's rank sum test; p** Mann- Whitney U test.

**Fig 3:**
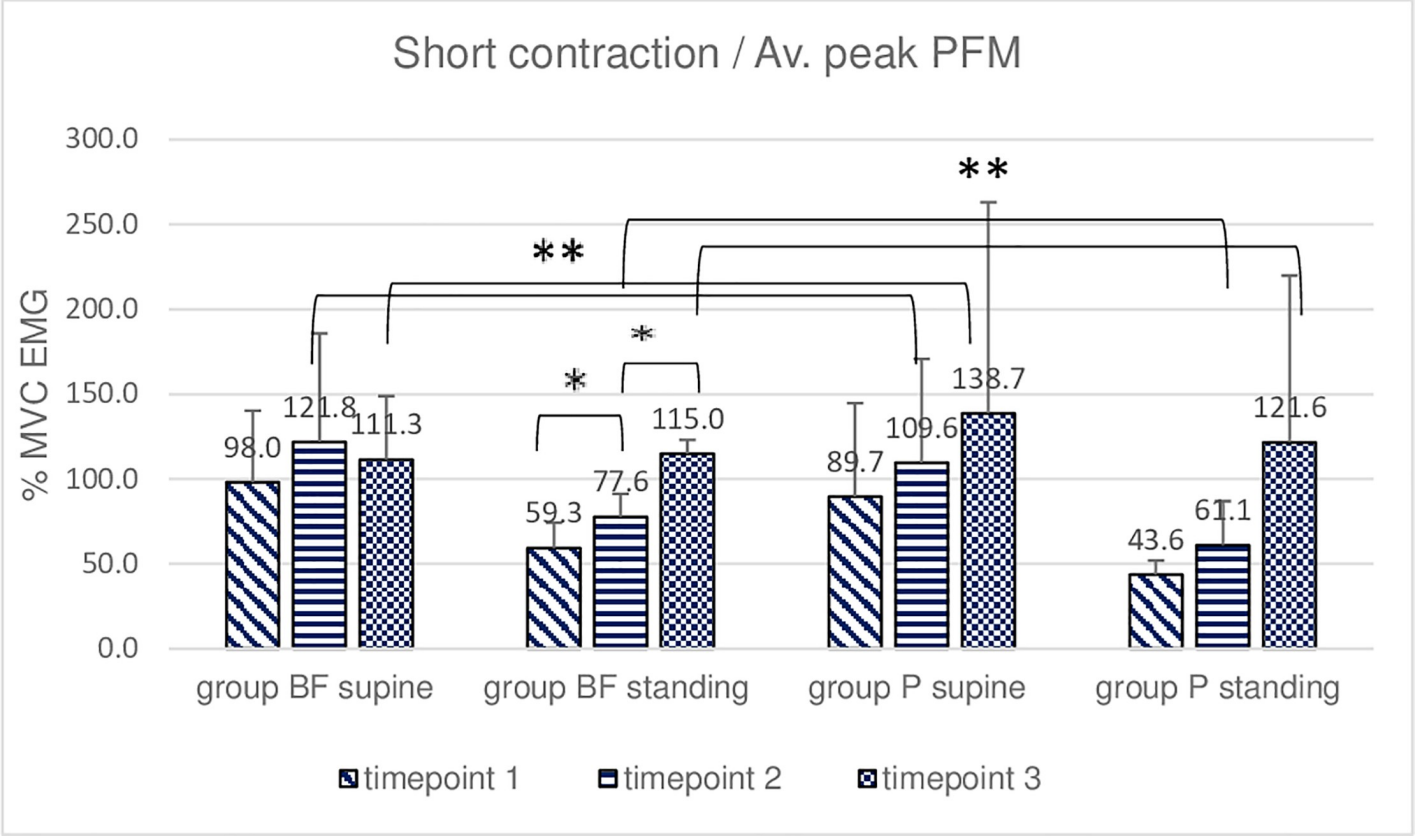
Intra- and intergroup analysis of sEMG average peak %MVC amplitudes of the short contraction of the pelvic floor muscles; p* Post-hoc Friedman's rank sum test.

**Fig 4:**
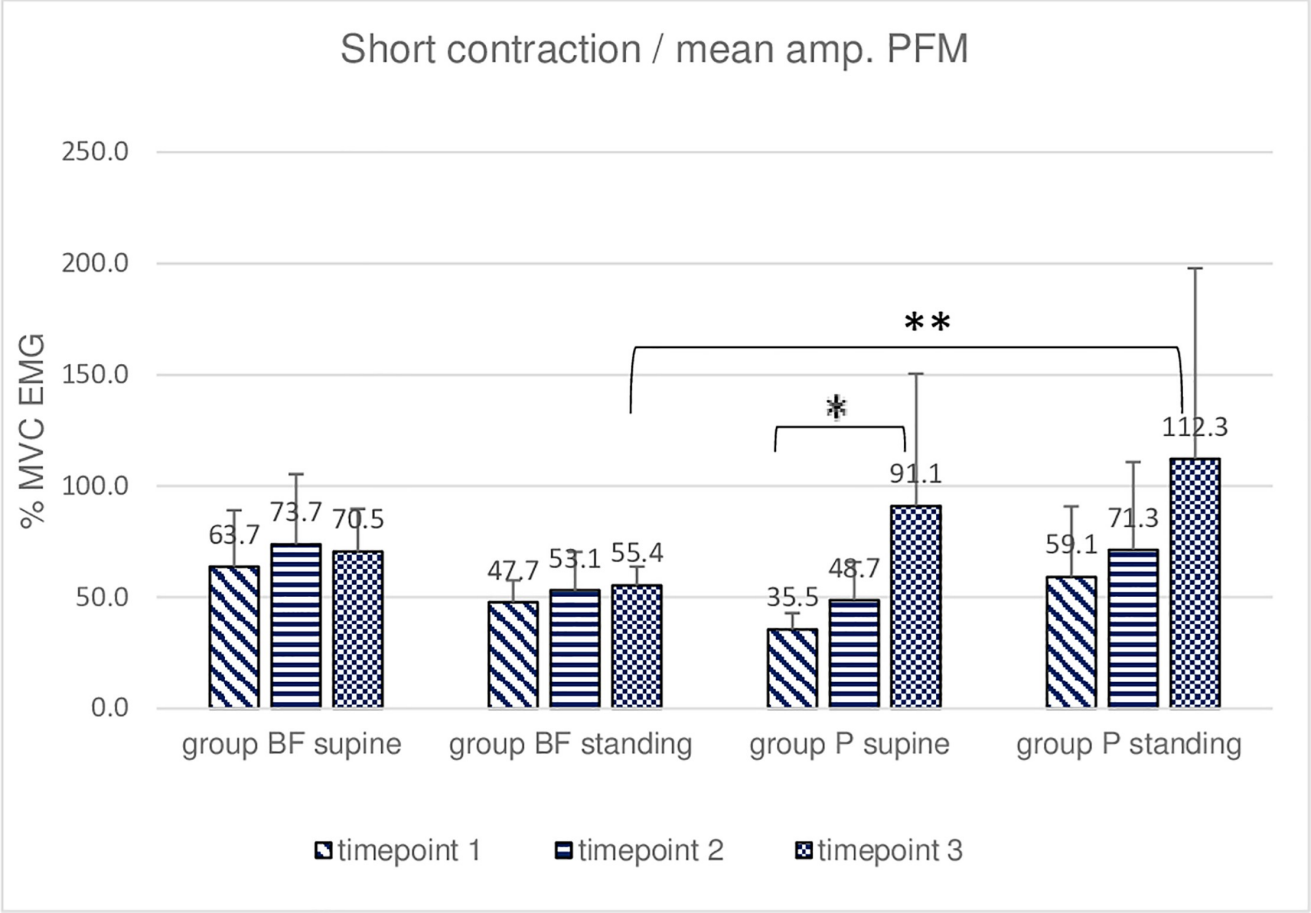
Intra- and intergroup analysis of sEMG mean %MVC amplitudes of the short contraction of the pelvic floor muscles; p* Post-hoc Friedman's rank sum test.

**Fig 5:**
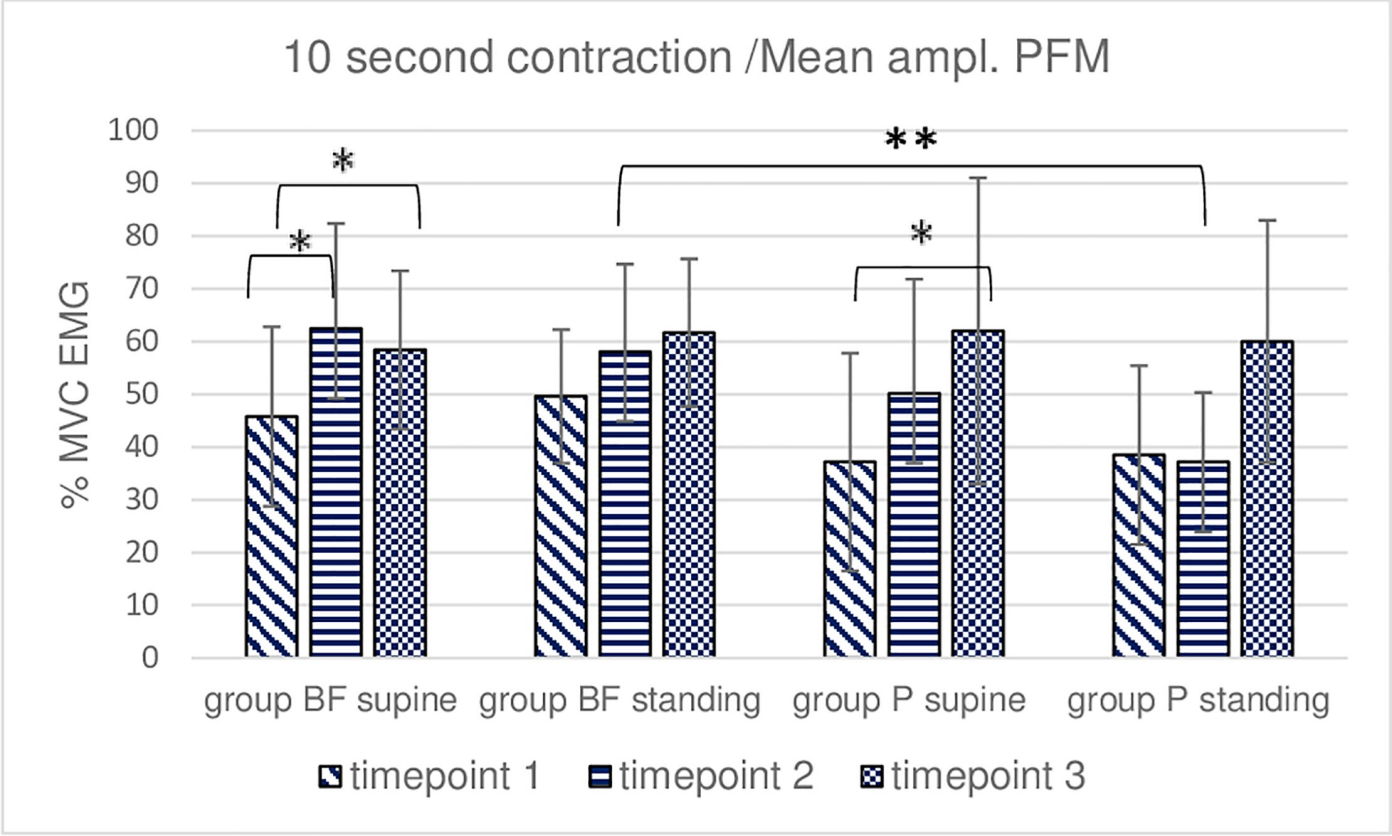
Intra- and intergroup analysis of sEMG mean %MVC amplitudes of the 5 repetitions of 10-second voluntary contraction of pelvic floor muscles; p* Post-hoc Friedman's rank sum test; p** Mann- Whitney U test.

**Fig 6:**
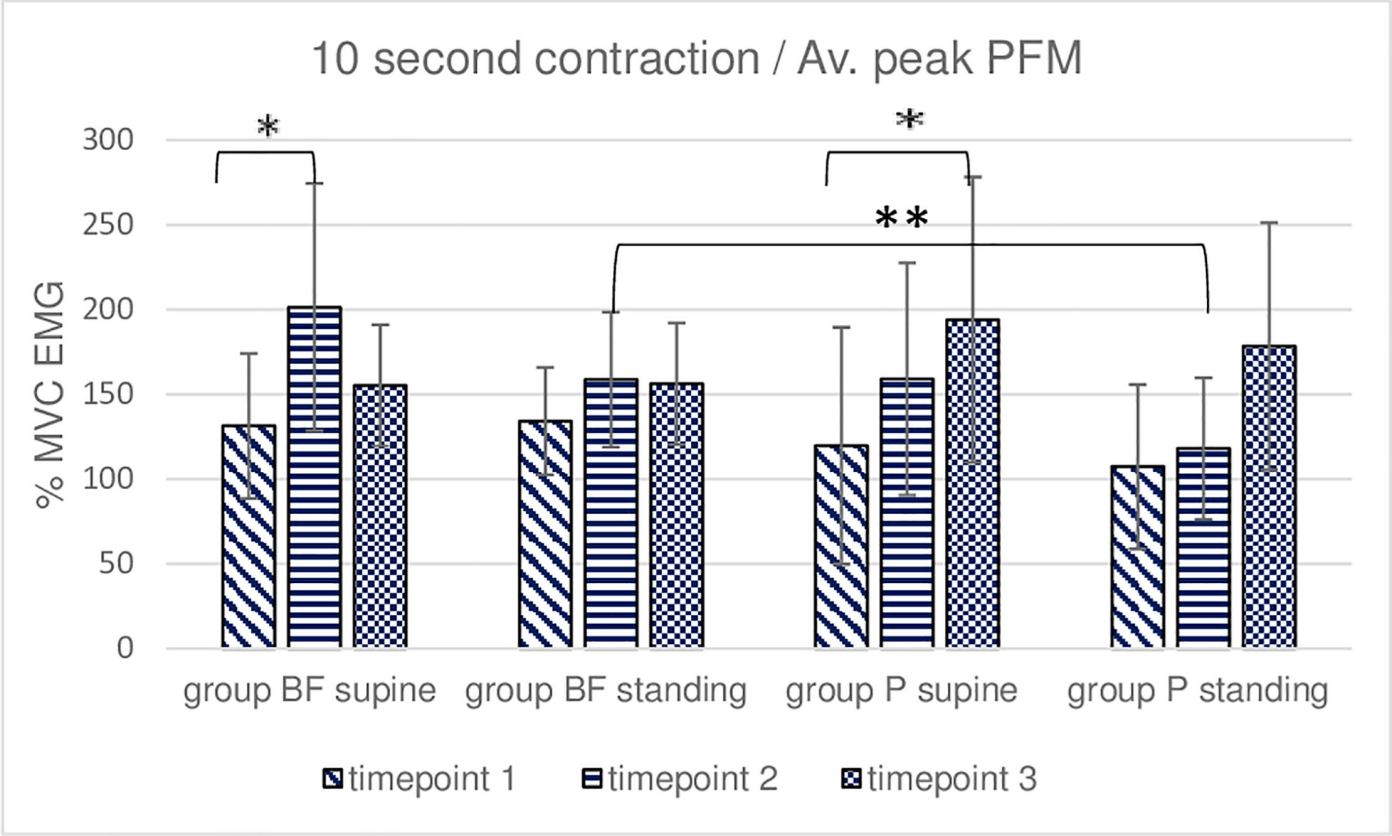
Intra- and intergroup analysis of sEMG average peak %MVC amplitudes of the 5 repetitions of 10-second voluntary contraction of pelvic floor muscles; p* Post-hoc Friedman's rank sum test; p** Mann- Whitney U test.

**Fig 7:**
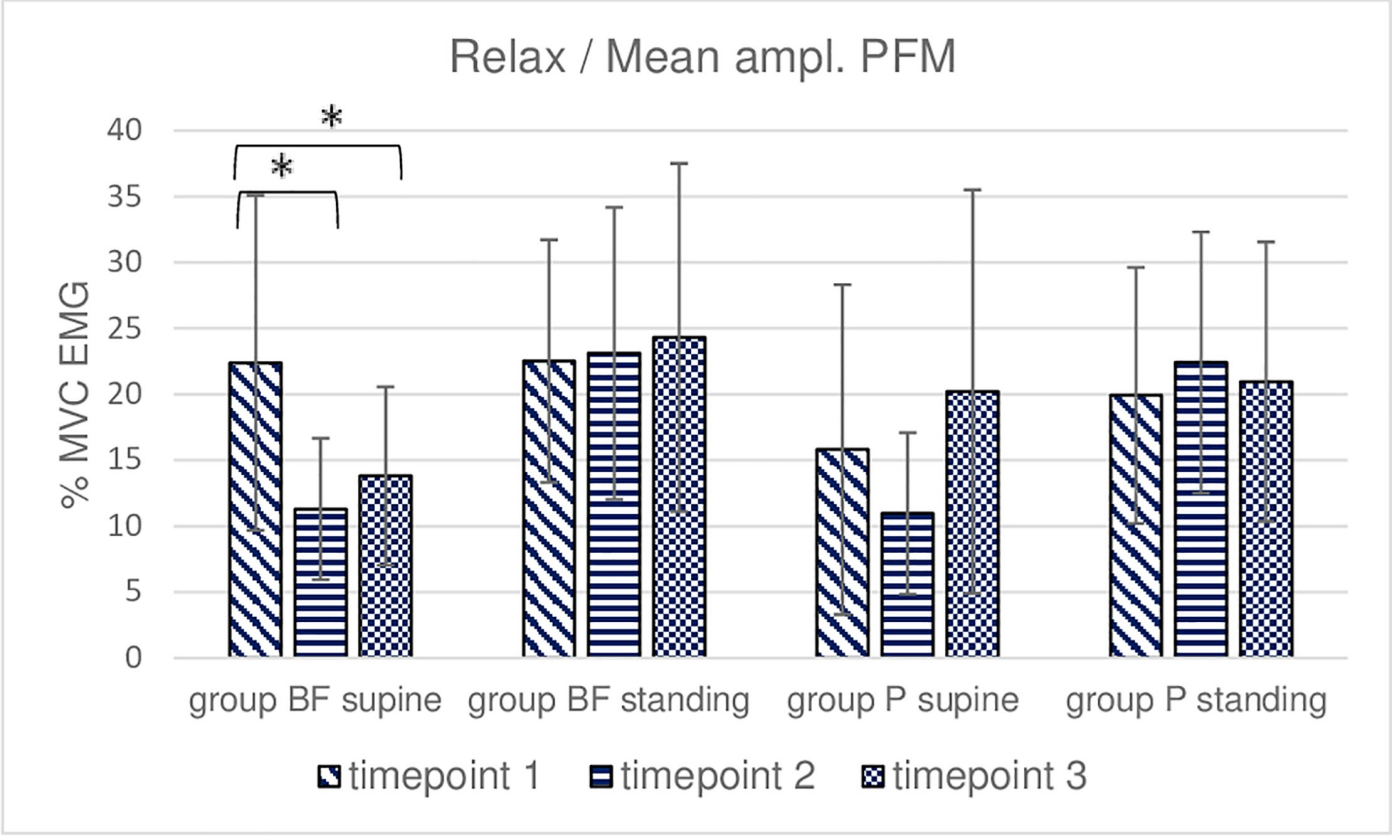
Intra- and intergroup analysis of sEMG mean %MVC amplitudes of the relaxation of the pelvic floor muscles; p* Post-hoc Friedman's rank sum test.

**Fig 8:**
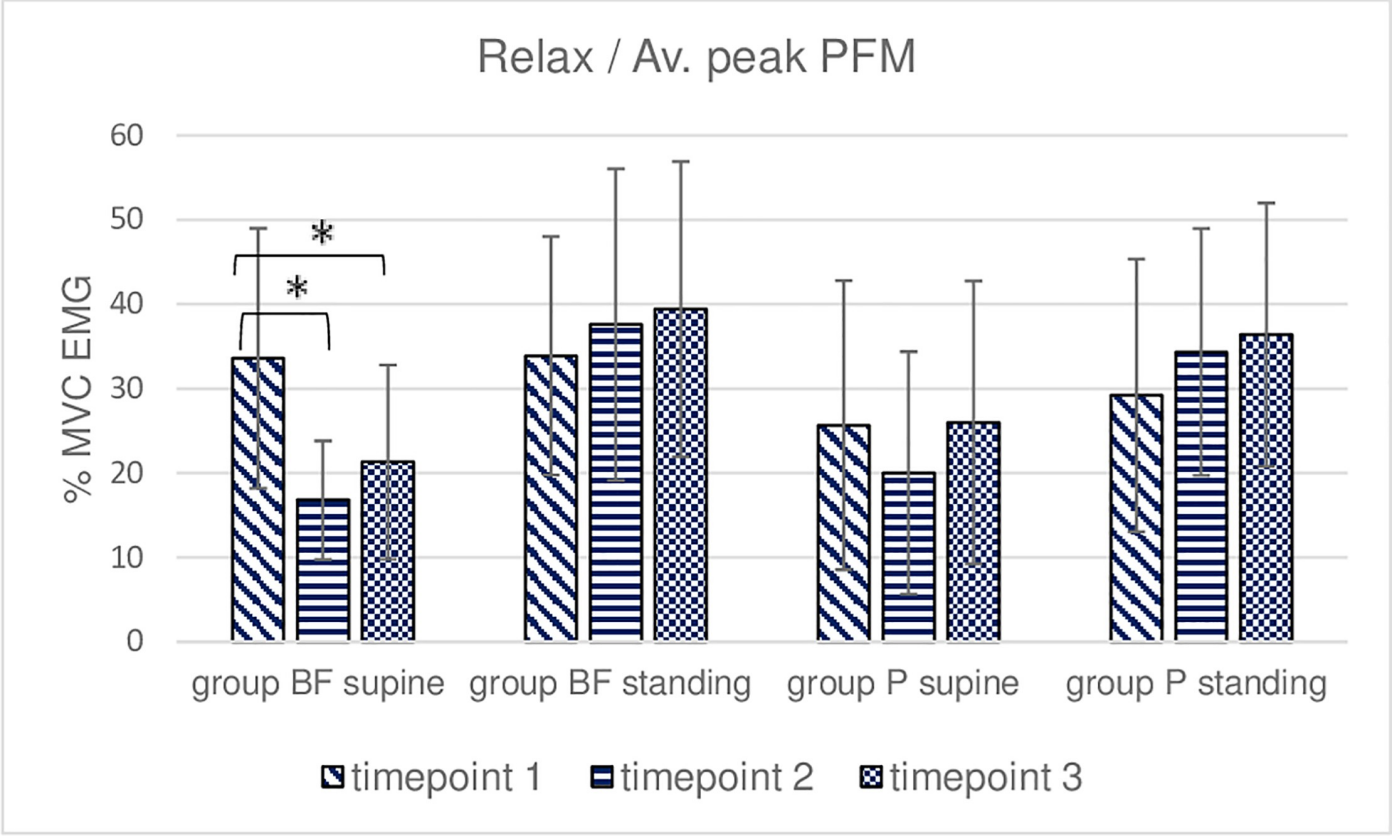
Intra- and intergroup analysis of sEMG average peak %MVC amplitudes of the relaxation of the pelvic floor muscles; p* Post-hoc Friedman's rank sum test.

The BF and P groups did not differ with respect to age, mean BMI, number of pregnancies (Table 1), mean number of urinary leakage episodes, mean micturition frequency, KHQ scores, severity of symptoms as assessed with the ICIQ-UI SF questionnaire (timepoint 1 in Table 2) and sEMG activity of pelvic floor muscles (timepoint 1 in Figs 1 to 8).

**Table 1:** Summary of characteristics of study participants.

Characteristics	BF group n = 18	P group n = 13	p-value
	mean ± SD or n (%)	mean ± SD or n (%)	
Age	52.9 ± 4	51.5–6 ± 5.2	0.57*
BMI	24.5 ± 3.04	23.7 ± 2.6	0.41*
BMI <30kg/m^2^	17 (94.44%)	13 (100%)	0.5**
BMI > 30kg/m^2^< 33kg/m^2^	1 (5.56%)	0 (0%)	0.5**
Number of pregnancies			
none	0	1 (7.7%)	0.2**
one time	3 (16.67%)	1 (7.7%)	0.45**
two time	9 (50%)	6 (46.1%)	0.83**
three time	5 (27.78%)	4 (30.8%)	0.79**
four time	1 (5.55%)	1 (7.7%)	0.89**
Cesarean section			
none	13 (72.22%)	11 (84.6%)	0.4**
one time	3 (16.67%)	1 (7.7%)	0.68**
two time	2 (11.11%)	1 (7.7%)	0.81**
Vaginal delivery			
none	2 (11,11%)	1 (7.7%)	0.75**
one time	4 (22.22%)	2 (15.4%)	0.63**
Two time	7 (38.8%)	7 (53.8%)	0.41**
three time	5 (27.7%)	2 (15.4%)	0.4**
four time	0 (0%)	1 (7.7%)	0.2**
Current hormonal therapy			
no	14 (77.78%)	8 (61.5%)	0.33**
yes	4 (22.22%)	5 (38.5%)	0.33**

* U Mann Whitney test

** Chi square test (χ^2^)

**Table 2:** Comparison of symptom severity (based on voiding diaries) and quality of life at three measurement time points.

	Group BF							Group P							p**intergroup BF vs. P		
Variables	1	2	3	p* 1,*2*,*3*	*p* *1 vs 2*	*p* *1 vs 3*	*p* *2 vs 3*	1	2	3	p* *1*,*2*,*3*	*p* *1 vs 2*	*p* *1 vs 3*	*p* *2 vs 3*	1	2	3
Micturition frequency Mean ± SD	8.09 ± 2.71	6.76 ± 1.68	6.96 ± 1.2	0.023	>0.05	0.04	>0.05	7.67 ± 2.61	7.26 ± 1.8	6.95 ± 1.9	0.67	>0.05	>0.05	>0.05	0.56	0.54	0.6
Mdn	7.83	7	7.31					7	7	6.67							
Q1	6.08	5.83	6.08					5.33	6.67	6							
Q3	9.53	7.92	8					8.67	8.33	7							
Nocturia frequency Mean ± SD	0.65 ± 0.51	0.39 ± 0.32	0.23 ± 0.21	0.002	>0.05	0.02	>0.05	0.51 ± 3.89	0.25 ± 0.27	0.23 ± 0.23	0.032	>0.05	>0.05	>0.05	0.51	0.37	0.62
Mdn	0.33	0.33	0.12					0.33	0.33	0							
Q1	0.33	0	0					0.33	0	0							
Q3	1	0.67	0.33					1	0.33	0.33							
Incontinence episodes Mean ± SD	1.78 ± 1.01	0.56 ± 0.76	0.19 ± 0.28	<0.0001	0.01	0.01	>0.05	1.69 ± 1	0.36 ± 0.37	0.23 ± 0.56	<0.001	0.01	0.01	>0.05	0.8	0.73	0.46
Mdn	2	0.333	0					1.67	0.33	0							
Q1	0.75	0	0					1	0	0							
Q3	2.33	0.67	0.33					2.33	0.67	0							
part 1 KHQ score the sum of questions 1 & 2; Mean ± SD	108.24 ± 41.6	73.15 ± 40.17	64.706 ± 24.5	0.0008	0.05	0.01	>0.05	93.75 ± 38.12	51.39 ± 26.6	40.97 ± 24.5	0.004	>0.05	0.02	>0.05	0.43	0.08	0.02
Mdn	91.67	58.33	58.33					87.5	58.33	33.33							
Q1	83.33	58.33	58.33					58.33	33.33	25							
Q3	147.92	89.58	83.33					100	58.33	58.33							
part 2 KHQ score the sum of questions 3 to 8; Mean ± SD	180.01 ± 91.2	156.79 ± 85.05	144.248 ± 78.67	p<0.019	>0.05	0.01	>0.05	123.46 ± 48.56	76.62 ± 40.22	63.97 ± 37.8	0.017	>0.05	0.03	>0.05	0.09	0.003	0.0009
Mdn	151.25	136.11	138.89					121.33	66.67	64.39							
Q1	121.39	91.67	92.36					97.22	56.25	14.58							
Q3	198.25	219.44	174.31					142.36	105.56	100							
ICIQ -SF Mean ± SD	12.83 ± 3.68	8.56 ± 4.03	7.39 ± 2.38	0.00065	0.03	0.01	>0.05	11.73 ± 4.01	6.73 ± 2.76	6.07 ± 3.06	p<0.001	0.02	0.01	>0.05	0.34	0.19	0.09
Mdn	13.5	8	8					12	6	6							
Q1	12	5.25	5.5					8.5	5	4.5							
Q3	14.75	12	9					14	7	6.5							

Mdn–median, Q1—lower quartile, Q3- upper quartile, KHQ- King's Health Quality of Life Questionnaire, ICIQ—The International Consultation on Incontinence Questionnaire Urinary Incontinence Short Form

p* (1, 2, 3)—Friedman's ANOVA, p *1 vs*. *2*, *1 vs*. *3*, *2 vs*. *3—*Post-hoc Friedman's rank sum test

p** Mann- Whitney U test.

In the BF group, the post hoc Friedman rank sum test revealed statistically significant differences in mean micturition frequency, nocturia frequency and KHQ Part 2 scores before (timepoint 1) and 6 months after training (timepoint 3). Also, the mean number of incontinent episodes and KHQ Part 1 scores were significantly different at baseline and after training (timepoint 1 vs. timepoint 2) as well as at baseline and six month follow-up (timepoint 3). The ICIQ–UI SF scores were significantly reduced after training (timepoint 1 vs. timepoint 2) as well as at baseline compared to six -month follow up (timepoint 1 vs. timepoint 3) (Table 2).

In the P group, the post hoc Friedman rank sum test did not reveal statistically significant differences in micturition and nocturia frequencies. The number of incontinent episodes was significantly different at baseline and after training (timepoint 1 vs. timepoint 2) as well as at baseline and six -month follow-up (timepoint 1 vs. timepoint 3). The KHQ Part 1 and 2 scores were significantly different at baseline and 6-month follow-up (timepoint 1 vs. timepoint 3). A significant decrease was noted between the baseline and post-Pilates ICIQ–UI SF scores (timepoint 1 vs. timepoint 2) as well as between the baseline and six -month follow up (timepoint 1 vs. timepoint 3) (Table 2). No differences were revealed between the BF and P groups in micturition frequency, number of incontinence episodes and ICIQ–UI SF scores at any of the three timepoints. However, intergroup differences were noted regarding the KHQ-Part 2 scores immediately after training (timepoint 2) and at six -month follow-up (timepoint 3). The KHQ-1 scores proved better for the Pilates group at six -month follow-up (timepoint 3) (Table 2).

A statistically significant decrease in PFM resting activity was noted in the BF group between timepoint 1 and timepoint 2 (average peak and mean amplitude, 10-second resting activity trial in supine position). No such decrease was revealed by the post hoc Friedman rank sum test in the P group. A comparison of sEMG amplitudes using the same test demonstrated significant differences between supine and standing positions in both groups. Average peak and mean amplitude in supine and standing positions were higher in the P group at timepoints 2 and 3 (10-second resting activity trial, Figs 1 and 2).

The post-hoc analysis of the short contraction in the BF group did not reveal significant differences between the peak amplitudes measured at three-time points in the supine position and between the mean amplitudes in the same position. Significant differences were observed between peak amplitudes measured at timepoint 1 and timepoint 3 as well as timepoint 2 and timepoint 3 in the standing position (Fig 3). The post-hoc analysis in the P group did not reveal significant differences between the peak amplitudes measured at three-time points in the supine position. Mean amplitudes in the same (i.e., supine) position differed significantly at timepoints 1 and 3 (Fig 4). Significant differences were also observed between peak amplitudes measured at timepoint 1 vs. timepoint 3 in the standing position. Intragroup (post hoc Friedman rank sum test) comparison of sEMG amplitudes revealed differences between standing and supine-lying at baseline and timepoint 2 in group BF. No significant intergroup differences were revealed in short contraction trials in the supine position. Intergroup differences were seen at timepoint 2 in standing position for peak sEMG amplitude and at timepoint 3 for mean sEMG amplitude (Figs 3 and 4).

In the BF group, the post hoc Friedman rank sum test demonstrated significant differences in the mean (Fig 5) and peak (Fig 6) sEMG amplitudes recorded during 5 repeated 10-second voluntary PFM contractions between 1 and 2 timepoints and in the mean amplitude between 1 and 3 timepoints–all in supine-lying. In the P group, the post hoc test showed significant differences in the mean (Fig 5) and peak (Fig 6) sEMG amplitudes recorded during 5 repeated 10-second voluntary PFM contractions between 1 and 3 timepoints in supine-lying. No significant intergroup differences were revealed in 10-second contraction trial in the supine position. Intergroup differences were noted at timepoint 2 in standing position regarding peak and mean sEMG amplitudes (Figs 5 and 6).

Measurements performed during a 10-second relaxation trial in the BF group revealed a significant decrease in mean amplitudes and peak amplitudes between timepoints 1 and 2 as well as timepoints 1 and 3 in supine–lying (Figs 7 and 8). A difference was also noted between the study positions at timepoints 2 and 3. The post-hoc analysis of group P data did not reveal significant differences between measurements at all three timepoints with the exception of mean amplitude measurements at timepoint 2 (standing vs supine). No intergroup differences were found with respect to the average peak and mean amplitude in the 10-second relaxation trial (Figs 7 and 8).

No significant differences were noted in both groups regarding median frequency, mean frequency and RMS sEMG obtained during sustained voluntary PFM contractions in standing and supine-lying. The post hoc Friedman rank sum test revealed differences in BF’s median frequency between the standing and supine positions (baseline vs. timepoint 2) and mean frequency between the standing and supine positions (timepoint 2 vs. timepoint 3). The same test carried out in the P group demonstrated differences in median frequency and mean frequency between standing and supine positions (baseline vs. timepoint 2). Intergroup differences were noted with respect to median frequency and mean frequency during sustained voluntary PFM contractions in standing position (timepoint 2 vs. timepoint 3) (Table 3).

**Table 3:** Intra- and intergroup analysis of sEMG amplitude of the sustained 60-second contraction of the pelvic floor muscles.

	group BF sustained contraction			group P sustained contraction			p** intergroup BF vs P		
Positions	median frequency (Hz) ± SD	mean frequency (Hz) ± SD	RMS EMG (μV)± SD	median frequency (Hz) ± SD	mean frequency (Hz) ± SD	RMS EMG (μV)± SD	median frequency (Hz) ± SD	mean frequency (Hz) ± SD	RMS EMG (μV)± SD
supine 1	75.7 ± 15.4	90.6 ± 19.1	12.7 ± 9.7	76.7 ± 10.2	96.5 ± 13.9	12.8 ± 8	0.9	0.3	0.6
supine 2	72.84 ± 13.5	88.7 ± 14.3	15.1 ± 7.4	79.2 ± 15.5	96.2 ± 18.9	17.1 ± 8.2	0.3	0.2	0.6
supine 3	72.2± 13.1	87.5 ± 15.3	15.4 ± 6.5	74.4 ± 19.7	90.7 ± 20.42	15.9 ± 9.1	0.7	0.6	0.8
standing 1	61.26 ± 12.66	76.46 ± 16.02	14.9 ± 9.4	59.2 ± 11.21	75.3 ± 12.26	16.7 ± 7	0.6	0.4	0.3
standing 2	55.12 ± 12.92	69.9.59 ± 12.1	17.36 ± 8.17	67.92 ± 16.5	83.7 ± 17.3	21.5 ± 9.7	0.02	0.03	0.3
standing 3	56.47± 6.42	71.6 ± 8.19	18.41 ± 9.5	62.6 ± 5.7	77.35 ± 5.81	22.2 ± 9.4	0.01	0.02	0.3
*supine* p** *(1*,*2*,*3)*	>0.05	>0.05	>0.05	>0.05	>0.05	>0.05			
*supine p (1 vs*. *2)*	>0.05	>0.05	>0.05	>0.05	>0.05	>0.05			
*supine p (1 vs*. *3)*	>0.05	>0.05	>0.05	>0.05	>0.05	>0.05			
*supine p (2 vs*. *3)*	>0.05	>0.05	>0.05	>0.05	>0.05	>0.05			
*standing* p* (1,*2*,*3)*	>0.05	>0.05	>0.05	>0.05	>0.05	>0.05			
*standing p (1 vs*. *2)*	>0.05	>0.05	>0.05	>0.05	>0.05	>0.05			
*standing p (1 vs*. *3)*	>0.05	>0.05	>0.05	>0.05	>0.05	>0.05			
*standing p (2 vs*. *3)*	>0.05	>0.05	>0.05	>0.05	>0.05	>0.05			
*p (supine 1 vs*. *standing 1)*	0.05	>0.05	>0.05	0.01	0.01	>0.05			
*p (supine 2 vs*. *standing 2)*	0.01	0.01	>0.05	0.04	0.01	>0.05			
*p (supine 3 vs*. *standing 3)*	>0.05	0.02	>0.05	>0.05	>0.05	>0.05			

p* (1 2 3)—Friedman's ANOVA

p—Post-hoc Friedman's rank sum test

p** Mann-Whitney U test

**Fig 9:**
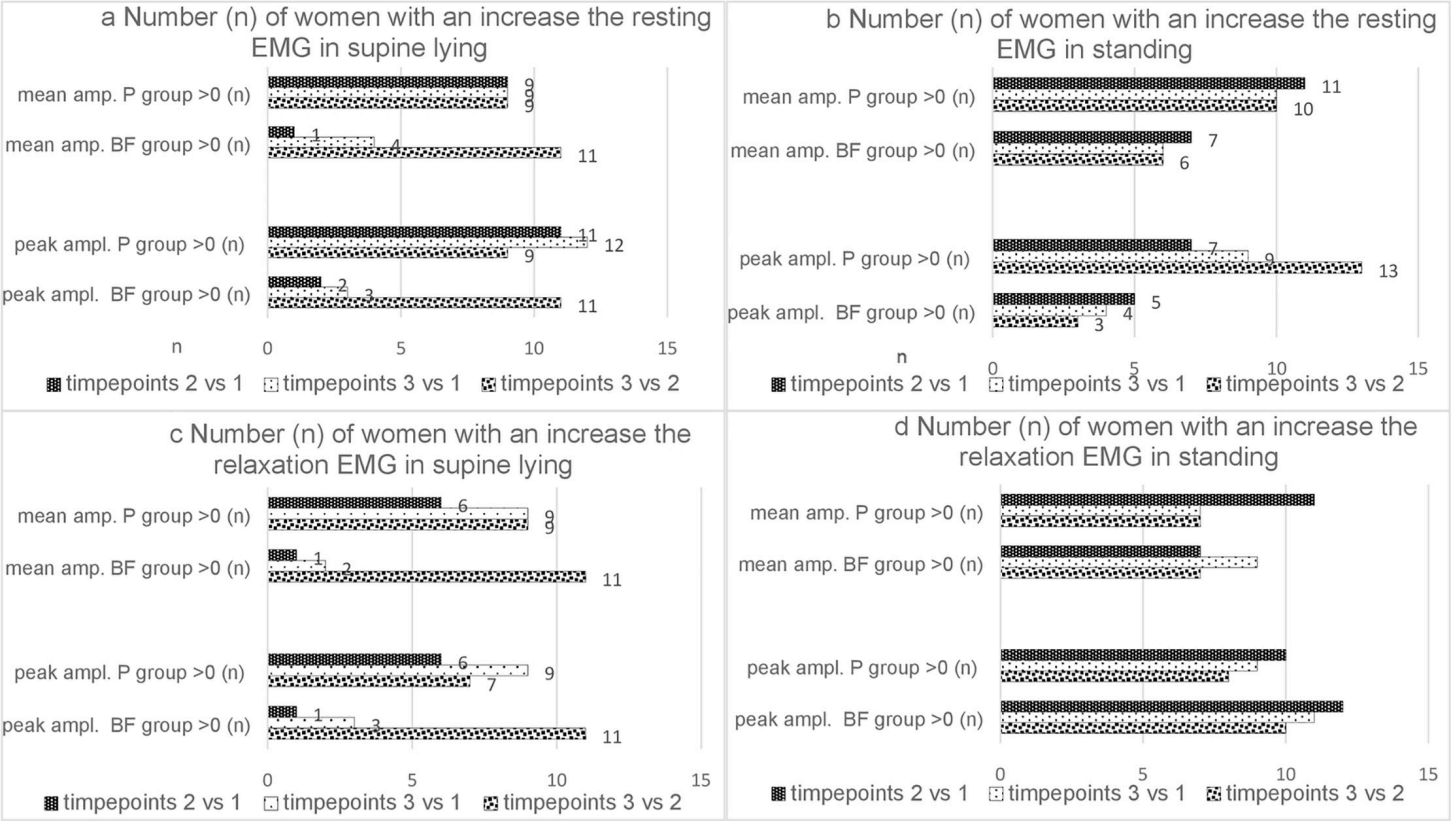
**a-d. Intra- and intergroup analysis the number of women with an increase (>0 (n)) in the resting activity and 10-second relaxation sEMG;** p Chi square test (χ^2^).

We compared the numbers of women whose sEMG amplitudes of the resting and relaxation trials increased or decreased between measurement timepoints 1, 2 and 3. Compared to the BF, the Pilates group included a significantly higher number of women who exhibited an increase in sEMG amplitude (peak, mean amplitudes during resting trials) after eight week trials as well as at six -month follow–up in both positions. The number of BF and P participants whose mean and peak amplitudes in supine–lying during resting trial increased between timepoint 2 and timepoint 3 (3 vs.2) was comparable in both groups (Fig 9A and 9B). The number of Pilates participants whose 10-second relaxation supine lying sEMG amplitudes (peak, mean) increased from timepoint 1 to timepoint 2 and timepoint 1 to timepoint 3 at 8-week and follow-up trials, was significantly higher compared to the BF. The number of BF and P participants whose mean and peak amplitudes in supine–lying increased between timepoint 2 and timepoint 3 was comparable in both groups (Fig 9C and 9D).

## Discussion

A few statistically significant differences were observed at the three measurement timepoints in the BF and P groups regarding normalized parameters of sEMG amplitude. Hence we concluded that an increase in sEMG amplitude parameters noted in the short contraction and 10-second contractions could not be unanimously attributed to the training programs. The obtained results did not demonstrate superiority of any of the two methods with respect to bioelectrical activity of the PFM. Also, no intergroup differences were observed regarding the relationship between median frequency, mean frequency and RMS EMG during sustained 60-second PFM contraction. Intragroup comparison only revealed differences in measurements obtained in the standing position. It would be interesting to further examine why eight weeks of regular pelvic floor muscle training with sEMG biofeedback did not increase the sEMG activity of the pelvic floor muscles, which contrasts with the findings of other studies [11, 13, 14, 15, 38, 39]. It seems likely that eight weeks of training were insufficient to induce changes in the physiology of muscle contraction. The literature does not provide an exercise protocol stating the optimal duration of the training, the number of contractions, number of contraction series, or the body position for sEMG-assisted biofeedback exercises in stress urinary incontinence. Bø and Sherburn have concluded that exercising can significantly improve the pelvic floor muscles in women provided that well-designed training is enhanced by appropriate monitoring and feedback [40] The optimal parameter selection for the pelvic floor muscle training is based on the protocol of muscle training of American College of Sports Medicine [41]. However, one should take into account that this optimization has been formulated for healthy adults.

Our protocol of pelvic floor muscle training with biofeedback is similar to those applied by other researchers regarding training duration (8 weeks), frequency of training sessions (3 times a week), the duration of quick (3seconds) and sustained contraction (up to 10 seconds) [12, 38, 42, 43] and the length of a training session (40 minutes) [15, 42]. However, the total of contractions per session differs between studies with protocols comprising equal numbers of phasic and tonic contractions, e.g., 30 [12, 38, 39, 43]. Our study proposes the following number of contractions per session: 21–60 for phasic and 45–120 for tonic contractions. We applied the same approach as the one adopted by Aukee, where the activity of the participant’s pelvic floor muscle was tested at baseline, before and after training in both the supine and standing positions [13, 14]. Exercise position during a test is important as standing is associated with increased intra-abdominal pressure leading to involuntary contraction of the PFMs [36]. The results of Bertotto et al. obtained in postmenopausal women with stress urinary incontinence have shown that eight sessions of pelvic floor muscle training with sEMG-assisted biofeedback resulted in increased myoelectric activity [12].

According to other studies, the sEMG-assisted biofeedback is associated with a significant increase in sEMG amplitude recorded during tests. Aukee et al. [13] obtained greater improvements in patients who did sEMG-assisted biofeedback training compared with those who only received the pelvic floor muscle training. The authors reported increased pelvic floor muscle activity (microvolts) and fewer leakage episodes.

Dannecker et al. [11] sought to determine the effect of 12 weeks of sEMG-biofeedback-assisted training on the PFMs in a study involving 390 women with stress (80%) and mixed (20%) urinary incontinence. The sEMG electric potential of the PFMs rose from a mean of 11.3 to 22μV, thus confirming the high effectiveness of PFM training.

Rett et al. [38] examined 26 women of reproductive age with SUI, finding them to have significantly better pelvic floor muscle strength (the Oxford scale) and sEMG amplitudes (μV) of all (tonic and phasic) contractions after 12 biofeedback sessions. In the study by Yoo et al. [29], the change in the average amplitude of tonic contraction between baseline and after the 8^th^ session, as measured by vaginal sEMG activity, was the only independent predictive factor of a successful response in the multiple logistic regression analysis. Biofeedback-assisted pelvic floor muscle training was successful in 57% of women with urinary incontinence.

A comparison of sEMG signal between different days or between different subjects should be made following the signal’s normalization [44]. The most used is the normalization to maximum voluntary contraction [33] and this we did. The lack of signal normalization in the above cited studies precludes comparison of the obtained results.

Considering the results of Naess et al. [45], who reported the reduction in hypertonicity of the pelvic floor muscles following three maximal voluntary contractions, the fact of an increase in the resting activity of the PFM in our Pilates group comes as a surprise.

We have not come across a study aiming to evaluate Pilates-induced changes in bioelectrical PFM activity. The knowledge on the effect of Pilates exercises on PFM function is still scarce. No differences in PFM functionality were noted between Pilates-practising and sedentary women [46]. It was also observed that Pilates instructors had urinary incontinence [47]. Several components of Pilates training might cause intra-abdominal pressure elevation and hence increased compression on the structures of the lesser pelvis and pelvic floor, ultimately leading to or aggravating urinary incontinence. This was not confirmed by Coleman et al. [48], who compared intra–abdominal pressure generated during Pilates Reformer and Mat roll-ups to that generated in sit-to-stand exercises. No statistically significant differences in mean max intra-abdominal pressure (IAP) were found between sit-to-stand and any of the Mat or Reformer exercises. Nevertheless, it turned out that twenty-five percent of the participants exceeded their individual mean max IAP sit-to-stand thresholds for the Reformer roll-up while thirteen percent did so during the Mat roll-up. Thus, Pilates exercises and modifications thereof should be carefully selected and sessions should be run by certified instructors. Also, exercisers should be observed during the training to help avoid extra load on the PFM.

Lausen et al. [49] reported the benefits of a 6-week course of 1-hour modified Pilates classes as an adjunct to standard physiotherapy for urinary incontinence. The classes involved slow, controlled movements focusing on posture and breathing. The study participants showed improvement in self-esteem, normal daily activities and personal relationships. Dias et al. [50] concluded that Pilates exercise program with PFM contraction performed by pregnant women was not capable of changing the PFM strength as assessed by the manometer. The other authors conclude that the effect of combining PFM contraction with hip isometric abduction or adduction does not have the claimed benefit for PFM function [51].

Our two groups exhibited different trends and variations in the recorded parameters during the resting activity and relaxation trials. Eight weeks of exercising seem to have induced both increases and decreases in sEMG amplitude in both groups. We analyzed the number of participants with increasing / decreasing amplitudes and made intergroup comparisons. The results are interesting. Resting EMG activity increased significantly following the Pilates program; consistently, the number of group P women with amplitude increase was greater compared to the BF group. This observation requires further evaluation. We hypothesize that BF women who monitored PFM activity during the biofeedback training were able to better control resting activity of these muscles.

## Conclusions

KHQ demonstrated that, as regards the quality of life, Pilates exercises turned out superior to sEMG biofeedback while the ICIQ-SF analysis indicated similar efficacy of both exercise programs. Intragroup improvements in micturition frequency, incontinence (leakage) episodes, and nocturia frequency were comparable.

Both sEMG biofeedback and Pilates exercises failed to cause a clear increase in bioelectrical PFM activity during contractions.

A decrease in bioelectrical PFM activity was noted at the resting activity trial and during relaxation after sustained contraction, but solely in BF’s supine-lying sEMG, which should be considered a beneficial effect of the training. No such effect was noted in the Pilates group. However, these observations do not support pelvic floor muscle training with sEMG biofeedback as more a favorable intervention in stress urinary incontinence.

## Limitation

Several limitations of our study should be pointed out. Narrow age-range of the study participants does not allow conclusions to be generalized across other age groups. Also, the study population should be larger as participants might be lost to the ultimate follow-up measurements. Another limitation is that no other method, e.g. perineometry, was used to evaluate the strength of the PFM. Finally, trial blinding was not possible due to the lack of centers for pelvic floor muscles rehabilitation in our country. All participants were examined in our laboratory throughout the study period.

## Supporting information

S1 Table Protocol for pilates program. (DOCX)

S1 Dataset (XLSX)
