# Supplementary material for: Electromyographic characteristics of pelvic floor muscles in women with stress urinary incontinence following sEMG-assisted biofeedback training and Pilates exercises
Source: PLoS One. 2019 Dec 2;14(12):e0225647. doi: 10.1371/journal.pone.0225647 (PMC6886793; doi:10.1371/journal.pone.0225647)
Supplement: S1 Table — (DOCX) [file pone.0225647.s001.docx]

S 1 Table Protocol for Pilates program

| Week | Exercise | Description |
| --- | --- | --- |
| 1 - 4 | Principles of breathing-movement coordination | Supine-lying. breathing exercises; diaphragmatic breathing with spine lengthening and neutral pelvis. Breath wringing out of pelvis bottom; learning the response of pelvic floor muscles to breathing |
|  | Pelvic clock | Supine-lying. Movements of the pelvis. Reference points: twelve o’clock – pubic bone; six o’clock – belly button; nine o’clock – left anterior superior iliac spine; three o’clock – right anterior superior iliac spine. Roll the pelvis through all clock positions; coordination with appropriate breathing phase |
|  | Side kick | Side lying. Lower limbs flexed to 90° at the hip and knee, ankles in dorsal flexion. Exhale - leg up; inhale – hip extension; exhale – bend at your hip (x 10). Switch sides |
|  | Clams | Side lying. Bend your knees and thighs. Exhale – external rotation at the hip so that the top knee opens; feet together; inhale – leg back to the starting position (x 10). Switch sides |
|  | Hundred (for beginners) | Lie on your back, knee and hip forming 90-degree angles. Exhale – lift your knee in the air directly above your hips, hold the leg up for 10 full breaths (5 inhales and 5 exhales) maintaining proper breathing pattern and neutral spine position; leg back on the mat. Switch sides. |
|  | Swimming (for beginners) | Lie on your belly, hands under the forehead. Exhale – hip extension; inhale – return to the starting position. Keep alternating sides left leg / right leg |
|  | Spine twist | Sit up on a large gym ball. Exhale – twist your torso to the left; inhale – back to the starting position.  Repeat twisting to the right (10x) |
|  | Seated pelvic tilts | Sit tall. Inhale – tilt the pelvis forward. Exhale – tilt the pelvis back. |
|  | Balance | Standing position. Exhale - lift up on the toes. Exhale – slowly get back on your heels (x 10) |
|  | Shoulder Bridge | Lie on your back. Inhale to prepare. Exhale – roll through the spine and lift your hips; inhale – maintain the position; exhale – roll the spine back to the floor. Inhale – drop all the way down to your tailbone (x 10) |
| 5 - 8 | Balance on sensorimotor disc | Walk-in-Place; standing + arm circles.  Standing + Upper limbs abduction / adduction |
|  | Squats | Inhale – lower your hips; keep the spine straight and neutral pelvis. Exhale – return the hips to the starting position |
|  | Pelvic clock while sitting on a large gym ball) | Sliding the pelvis between reference points:  - pubic bone (twelve o’clock)  - coccygeal bone (six o’clock)  - right ischial tuberosity (three o’clock)  - left ischial tuberosity (9 o’clock);  inhale between 09.00 – 12.00 – 03.00 hours;  ex hale between 03.00 – 06.00 – 09.00 hours |
|  | Cat exercise | Get on all four limbs. Exhale – arch your back (= pelvic bottom closed). Inhale – return to neutral spine (= pelvic bottom open) |
|  | One leg circle | Side lying. Lift your top leg up slightly higher than the hips. Circle the leg with your pelvis in neutral position (x 10). Switch sides |
|  | Four point swimming | 4 point kneeling. Left hip extension; lift your left foot and stretch it backwards and upwards. Maintain a neutral pelvis (x 10). Switch sides |
|  | Single leg stretch | Lie on your back. Exhale – pick one knee up into tabletop position, right angle at hip and knee; inhale to prepare; exhale – straighten the leg. Inhale - knee back over the hip (x 10). Switch sides |
|  | Shoulder bridge - with a large ball | Lie on your back with a large ball under your legs. Exhale - articulate your spine all the way off the floor. Inhale – hold the position. Exhale and move your arms behind the head; exhale and place the arms along the trunk. Exhale and lower the hips (rolling the spine back to the floor).  Repeat 10 times |
|  | Plank (for beginners) | Lie flat down on your belly; forearms on the mat. Exhale – lift your torso and thighs off the floor; inhale – maintain the plank position; exhale – slowly come back to the resting position starting with the thighs, then pelvis and torso (x 10) |
|  | (Relaxation exercise – forest visualization) | Lie on your back or abdomen (whatever position is comfortable). Breathe consciously trying to visualize a forest |
